# Dr. Henry Flood

**Published:** 1914-02

**Authors:** 


Dr. Henry Flood, Bellevue, 1874, died at his home in Elmira, December 30, 1913, aged 60. Born at Lodi, he had been a resident of Elmira since childhood and was educated at the Elmira Free Academy, graduating in 1871. He began the study of medicine in the office of his father, the late Patrick H. Flood. In addition to his regular medical course, he studied in Vienna in 1878-9 and at the P. & S. (now Columbia) of New York, in 1889-90. Beside being prominent as a physician, he was mayor of Elmira in 1884-5 and was postmaster in 1889. He was one of the founders of the Elmira City Club. He had been sick for several months and had been confined to his room for six weeks, fully realizing and resigned to the fatal nature of his disability. Beside his widow and one son, Mr. Henry Flood of Poughkeepsie, he is survived by two nieces in the profession, Dr. Regina Flood Keyes of Buffalo and Dr. Mabel Flood, who had been associated with her uncle.

